# How the use of an online healthcare community affects the doctor-patient relationship: An empirical study in China

**DOI:** 10.3389/fpubh.2023.1145749

**Published:** 2023-04-06

**Authors:** Zhanyou Wang, Xin Zhang, Dongmei Han, Yaopei Zhao, Liang Ma, Feifei Hao

**Affiliations:** ^1^Management Science and Engineering, Shandong University of Finance and Economics, Jinan, China; ^2^School of Labor Relations, Shandong Management University, Jinan, China; ^3^School of Information Engineering, Shandong Management University, Jinan, China; ^4^Shandong Labor Vocational and Technical College, Jinan, China; ^5^College of Traditional Chinese Medicine, Shandong University of Traditional Chinese Medicine, Jinan, China

**Keywords:** online healthcare community, doctor–patient relationship, patients’ satisfaction, patients communicate, healthcare costs

## Abstract

Possible improvements to the doctor-patient relationship are an important subject confronting national healthcare policy and health institutions. In recent years, online healthcare communities have changed the ways in which doctors and patients communicate, especially during the COVID-19 pandemic. However, previous research on how usage of online healthcare communities has affected the doctor-patient relationship is rather limited. This paper proposes a research model to investigate the relationship between online healthcare community usage and the doctor-patient relationship. An analysis of 313 patients’ data using structural equation modeling showed the following. First, the use of an online healthcare community has a positive impact on doctor-patient communication, helps improve the performance of healthcare procedures, and reduces healthcare costs. Second, doctor-patient communication and healthcare costs have a positive impact on patients’ emotional dependence and patients’ perception of healthcare quality, while healthcare procedures do not have this impact. Finally, patients’ emotional dependence and perception of healthcare quality have a positive effect on doctor-patient relationship through the mediator of patients’ satisfaction.

## Introduction

The improvement of the doctor-patient relationship in the field of healthcare is an important issue that is currently troubling the country, including both doctors and patients (1). According to the 2020 National Big Data Report on Healthcare Damage Liability Dispute Cases, the total number of healthcare damage liability dispute cases in China was 18,670, up about 3 percent from 2019. At the same time, although China has carried out many healthcare reforms that have had some effect on the quality of healthcare services, the fundamentally tense relationship between doctor and patient remains largely unchanged. Patients complain that they do not receive proper services, and their interests can be harmed (2). For their part, doctors complain of the pressures put on them due to various social and economic aspects of providing healthcare (3). Therefore, means of improving the doctor-patient relationship in healthcare are urgently necessary.

Scholars have made attempts to improve the doctor-patient relationship, as seen in the literature. Studies have focused on patient satisfaction, trust, and communication between doctors (4, 5), the use of information systems, doctors’ caring attitude, interpersonal skills, treatment interpretation (1, 6), technical skills, hospital equipment, clinical ability, and others (5, 7). For example, Chenget al. (8) found that doctors’ caring attitude, interpersonal skills, treatment interpretation, technical skills, hospital equipment, and clinical competence had a significant positive impact on patient satisfaction and the improvement of doctor-patient relationship. Naidu (9) pointed out that patients’ perceptions, especially of doctors’ communication skills, were also important determinants of satisfaction and influenced the improvement of the doctor-patient relationship. Grunloh et al. (10) showed that patient loyalty is an important factor in patients’ willingness to repeatedly seek healthcare treatment. If healthcare and healthcare services can retain patients and make them loyal customers, it will improve the doctor-patient relationship and bring long-term commercial benefits to healthcare institutions.

Although previous studies have produced outstanding contributions to the doctor-patient relationship literature, few have explored how the use of online healthcare communities affects the doctor-patient relationship and how this effect comes about. In recent years, with the rapid development of the Internet, online healthcare communities have gained attention. These communities, which have patients or potential patients as their main service objects, provide health information, online consultations, healthcare consultations, and other services, providing channels for doctor-patient interactions through online community platforms (11). By examining the role that online healthcare communities play in the doctor-patient relationship, this study conducts a novel exploration of how the use of online healthcare communities by patients affects the doctor-patient relationship. Clarifying this issue is of great significance for understanding patients’ use level of online healthcare communities and to develop improvements in the service level of doctors in the online healthcare community.

In the course of this examination, this study constructs a theoretical model for the relationship between online healthcare community usage and the doctor-patient relationship. In this way, this study contributes to the doctor-patient relationship literature and by exploring the mediator role of doctor-patient communication, healthcare procedures, healthcare costs, emotional dependence, and perceptions of healthcare quality for online healthcare community usage and the doctor-patient relationship. Finally, this study provides practical insight for national healthcare and health institutions to improve the doctor-patient relationship.

## Research hypotheses and model proposal

### Online healthcare community usage and doctor-patient communication

Doctor-patient communication refers to the communication and exchange that patients and their families have with healthcare personnel in healthcare institutions and online healthcare communities regarding diagnosis and treatment, services, and health, psychological, and social factors (12). Wang et al. (11) found that doctor-patient communication includes six main sub-categories: emotional attitude, mode, adequacy, efficiency, content similarity, and communication service quality. The use of online healthcare communities has a positive impact on doctor-patient communication, as it can improve the degree of communication and patient satisfaction, as well as meeting patients’ expectations of consultation (11). Other studies have confirmed parts of these conclusions. For example, prior research finds that online doctor-patient communication can break through time–space limitations of traditional healthcare treatment situations and present new features and trends for doctor-patient communication practice (13). Patients can not only have private conversations with doctors anytime and anywhere to obtain healthcare services and health information with a higher degree of specialization and customization, but they can also have a stronger sense of control and participation in the process of doctor-patient communication with the empowerment of professional healthcare resources and healthcare information (14, 15). Therefore, online healthcare communities provide a platform for doctor-patient communication. When patients use an online healthcare community, the doctor-patient communication can be significantly improved. Based on this discussion, this paper proposes the following hypothesis:

*H1*: Use of an online healthcare community has a positive impact on doctor-patient communication.

### Online health community use, healthcare process, and healthcare costs

The healthcare treatment process refers to the steps and main procedures that patients need to go through to obtain healthcare treatment, falling into two sub-categories: offline healthcare treatment process and online healthcare treatment process (16). The use of an online healthcare community has a positive impact on the healthcare treatment process because online healthcare community provides online registration, which is much more convenient for patients (17). Overall, the use of an online healthcare community can shorten the time required for patients to see a doctor and optimize the healthcare treatment process through online registration and appointment of examination items (18).

Healthcare costs are the comprehensive expenditures of economy, time, energy, and other resources in the process of seeking healthcare treatment, which includes two main sub-categories: providing free consultation services online and reducing the cost of healthcare treatment online (19). In traditional healthcare treatment, patients have higher healthcare costs, especially with respect to transportation, accommodation, registration, waiting for healthcare treatment, payment, and other costs of long-distance healthcare treatment. However, online healthcare communities can alleviate these problems to a certain extent and have greater selectivity than traditional healthcare patients. In particular, the use of an online healthcare community can reduce the time and monetary costs of healthcare treatment (20–22). From this discussion, this paper proposes the following hypotheses:

*H2*: Use of an online healthcare community has a positive impact on the healthcare process.

*H3*: Use of an online healthcare community has a negative impact on healthcare costs.

### Doctor-patient communication, emotional dependence, and patients’ perception of healthcare quality

Online doctor-patient communication uses computer terminals or mobile devices as media, doctors and patients as interactive parties, and healthcare consultation or health exchange as communication purposes (12). This study examines online healthcare communities in which both doctors and patients participate. A survey has found that online doctor-patient communication often uses graphic consultation (14). Patients may generate emotional dependence and satisfaction while using online healthcare platform communication (2, 23). Emotional dependence refers to an outcome-based emotional evaluation generated by patients in their consumption of healthcare services, which reflects the emotional connections and bonds established among patients, doctors, and the platforms (24). Online healthcare platforms are a novel type of communication channel, allowing patients to feel that their doctor is a bit like themselves, similar to the concept of family doctor or private doctor in Europe and the United States. Online healthcare platforms allow patients to ask questions of their doctor and make it easier to form a relationship of trust and dependence between the patient and the doctor (5).

The doctor’s attitude is very important as well, especially if the doctor has a good attitude. The process of communicating with their doctor may bring psychological comfort to the patient; in such a case, the patient may feel that their condition is not as serious, which gives patients confidence. Doctor-patient communication based on online healthcare platforms have a positive impact on patients’ emotional dependence. Additionally, online healthcare platforms can improve the degree of communication, improve patients’ perception of healthcare quality, and meet patients’ expectations through consultation (8, 9). Taken together, these indicate that doctor-patient communication using an online healthcare platform can have a positive impact on patients’ perception of healthcare quality. From this discussion, the following hypotheses are proposed:

*H4*: Doctor-patient communication has a positive impact on patients' emotional dependence.

*H5*: Doctor-patient communication has a positive impact on patients' perception of healthcare quality.

### Healthcare process, healthcare costs, emotional dependence, and patients’ perception of healthcare quality

The healthcare process involving an online healthcare community includes online appointments and offline treatments. The healthcare process involving an online healthcare community can save healthcare expenses and have an impact on patients’ emotional dependence and satisfaction. For example, online healthcare community can save patients’ time in making an appointment and save them time in queuing for offline treatment (15). Online healthcare communities can save treatment costs, facilitate patients, influence them, and improve their emotional dependence and perception of healthcare quality (25).

The use of an online healthcare community can reduce the cost of healthcare treatments for patients, which may impact on patients’ emotional dependence and satisfaction. For example: using an online healthcare community is convenient for patients, increasing their emotional dependence (26). In addition, the use of an online healthcare community improves patient experience by reducing patient costs, thus affecting patients’ perception of healthcare quality (27). From this discussion, this paper proposes the following hypotheses:

*H6*: Improvement of healthcare process has a positive impact on patients' emotional dependence.

*H7*: Improvement of healthcare process has a positive impact on patients' perception of quality of care.

*H8*: Improvement of healthcare costs have a positive impact on patients' emotional dependence.

*H9*: Improvement of healthcare costs have a positive impact on patients' perception of healthcare quality.

### Patients’ emotional dependence, perceived healthcare quality, and doctor-patient satisfaction

Emotional dependence refers to outcome-based emotional evaluation that is generated by patients in their interaction with healthcare services and reflects the emotional connection and bond established between patients, doctors, and online healthcare platforms (28). The perception of healthcare quality refers to the quality and effects of healthcare services, largely, the timeliness, effectiveness, and safety of healthcare services (29). Patient satisfaction refers to patients’ satisfaction with the online healthcare community and the service level of their doctors (30). While using online healthcare communities, patients’ emotional dependence and perception of healthcare quality may affect their patient satisfaction (31). The use of an online healthcare community can increase the emotional dependence of patients, and when the patient generates emotional dependence on the doctor, indicating that the patient is satisfied with treatment by that doctor, patient satisfaction improves. On the other hand, as the patient perception of healthcare quality improves, the patient also has increased satisfaction because higher healthcare quality, which includes timeliness, effectiveness, and safety of healthcare services, generates greater patient satisfaction (32). This paper proposes the following hypotheses from this discussion:

*H10*: Patients' emotional dependence has a positive effect on patient satisfaction.

*H11*: Patients' perception of healthcare quality has a positive impact on patient satisfaction.

### Patient satisfaction and the doctor-patient relationship

Patient satisfaction plays an important role in the relationship between healthcare service quality and the doctor-patient relationship. Studies have shown that when patients are satisfied, they perceive higher healthcare quality and an improved doctor-patient relationship (33). According to Wang et al. (11), in the online healthcare community, patients can find a doctor at any time, and many patients already trust their doctor, who, through online communications, can reply to the patients in a timely fashion, increasing trust even more, ultimately improving the entire doctor-patient relationship. Trust in doctors may be associated with increased trust throughout the entire site of health care, such as a hospital, such that doctors in the hospital and some healthcare services provided by the hospital also become more trusted (34). Thus, patients will trust the hospital more after the experience of using an online healthcare community. In conclusion, the online healthcare community can improve the trust and satisfaction of patients and then improve the doctor-patient relationship as well (1, 23). From this discussion, this paper proposes the following hypothesis:

*H12*: Patient satisfaction has a positive impact on the doctor-patient relationship.

### Theoretical model proposed

**Figure 1:**
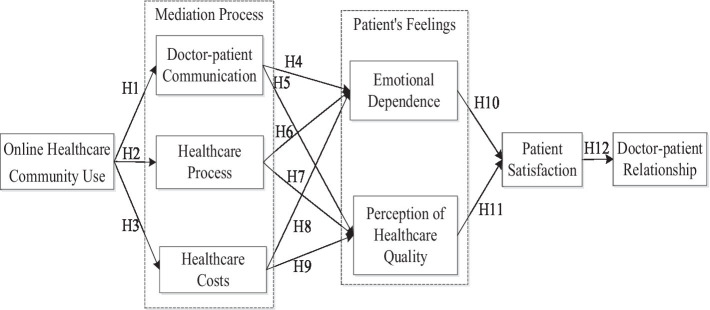
Theoretical model.

A theoretical model of the research is proposed as shown in Figure 1. The model explains how the use of an online healthcare community affects doctor-patient communication, healthcare treatment, and treatment cost, from this affecting patients’ emotional dependence and patients’ perceptions of healthcare quality, which ultimately has an impact on patient satisfaction and the doctor-patient relationship.

## Research design

### Measurement of variables

This paper collected data with a questionnaire survey. Question items and measures were drawn from earlier studies. Among these was doctor-patient communication, including five measure items, taken from Ha and Longnecker (13) and Wang et al. (11). The doctor-patient relationship was measured with included five measurement items revised from Zhang et al. (1). The scale of emotional dependence was adopted from Macia et al. (28) and Wang, Zhang, Han, and Ma (11). The measure of healthcare costs was adopted from Wang et al. (11). The measure for the healthcare process was revised from Jin et al. (35). The perception of healthcare quality was revised from Ramadan and Arafeh (36). Patient satisfaction measures were taken from Batbaatar et al. (37) and Chen et al. (23). Finally, online healthcare community use was revised from Goh et al. (38) and Liu et al. (39). The complete measurement items of the scale are shown in Table 1. The questionnaire was presented on a 7-point Likert scale ranging from 1(strongly disagree/very unlikely) to 7(strongly agree/very likely).

**Table 1:** Measurement items.

Dimension	Statements
Doctor-patient communication (DC)	Patients can communicate with their doctors in an online healthcare community
	Patients can see online healthcare doctors’ answers to patients’ questions
	Online healthcare communities can help patients communicate privacy issues with their doctors
	Online healthcare communities allow me to communicate with doctors without time and space constraints
	I feel I can communicate with doctors more fully by using the online healthcare community
Doctor-patient relationship (DPR)	After using the online healthcare community, I think my complaints about the hospital will decrease
	After using the online healthcare community, I think disputes between hospitals and patients will be reduced
	After using the online healthcare community, I feel that the doctor-patient relationship is less tense than before
	After using the online healthcare community, I think other patients will complain less about the hospital
	After using the online healthcare community, I feel that the healthcare resources are not so tight
Emotional dependence (ED)	Online healthcare communities can meet the psychological needs of patients
	Online healthcare communities can meet patients’ spiritual needs
	Doctors in online health communities are emotionally understanding and supportive
	The use of online healthcare communities can alleviate patients’ anxiety in time
	The online healthcare community enables patients to enjoy the VIP-level service experience of family doctors
Healthcare costs (MC)	I use online health communities to save time
	I use online health communities to save energy
	The use of online healthcare communities can save certain economic costs (reduced travel costs, consultation fees, etc.)
	Using online healthcare communities can reduce my visits to physical hospitals
	Online health communities can offer free consultations
Healthcare process (MP)	The online healthcare community offers an appointment service
	Online healthcare communities can save waiting times
	Online health communities can optimize the healthcare process
	As a continuation of the offline healthcare treatment model, the online healthcare community can provide perfect online services
	Online health communities do not bring me into direct conflict with my doctors
Perception of healthcare quality (MQ)	Healthcare services in online healthcare communities are quick
	The healthcare services provided by the online healthcare community are effective
	The health services provided by online health communities are safe
	The healthcare services of the online healthcare community are humanized
	The healthcare services provided by the online healthcare community are perfect
Patient satisfaction (PS)	I am happy with the doctors in the online healthcare community
	I am satisfied with the service of the online healthcare community
	I’m happy with the way the online healthcare community flows
	I love online healthcare communities
	In my opinion, an online healthcare community can shield various external factors from offline healthcare treatment and improve patient satisfaction (patient healthcare experience)
Online healthcare community use (SU)	I use the online healthcare community to ask questions
	I use the online healthcare community to make appointments
	I use the online healthcare community to look up the information
	I use online healthcare communities to quickly find medicines
	I use the online healthcare community to popularize healthcare knowledge

### Data collection

After the completion of the questionnaire design, this paper invited three associate professors and five graduate students to fill in the questionnaire to ensure its readability. This paper also integrated study instrument piloted among the population. This paper used the snowball sampling method and we distribute questionnaires in the WeChat group to collect the sample data. Each participant was asked about any prior experience using an online healthcare community and filled out a questionnaire to collect their personal information and personal experiences in the use of the online healthcare community platform. The questionnaire begins with a brief description of the purpose of the survey, namely, to explore the use of online healthcare communities. Following this, interviewees were required to provide basic personal information, including gender, age, education level, monthly income, and health status. Finally, interviewees were required to share their experience in using an online healthcare community platform and online healthcare treatment based on their own experience. A total of 315 respondents completed the survey. Among these, 313 were valid, with an effective completion rate of 99.36%.

### Data analysis tool

Smart PLS3.0 was used to analyze the data and test the hypotheses. A second-generation multivariate statistical analysis tool, PLS3.0 can evaluate both measurement models and structural models. A PLS model can use a smaller sample size than the structural equation model based on covariance, and PLS is not limited by the normal distribution, so it is more suitable for a study of this kind. Following a two-step analysis (40), the measurement model and the structural model were tested in this paper.

## Data analysis and hypothesis testing

### Descriptive statistical analysis

Table 2 lists the sample characteristics of the collected questionnaires. Most respondents were female, 57.51%. Most were between 41 and 50 years old, 66.13%. Most had a high school education or below, 58.15%. Most monthly incomes were less than 5,000 RMB, 67.41, and 36.10% were less than 3,000 RMB, and 31.31% were between 3,000 RMB and 4,999. Most were in good health, accounting for 38.98%.

**Table 2:** Descriptive statistics of interviewees’ characteristics.

Items	Attribute	Number	%
Gender	Male	133	42.49%
	Female	180	57.51%
Age	≤20 years old	6	1.92%
	21–30 years old	20	6.39%
	31–40 years old	42	13.42%
	41–50 years old	207	66.13%
	﹥50 years old	38	12.14%
Education	Senior middle school or below	182	58.15%
	Junior college	54	17.25%
	Bachelor’s degree	37	11.82%
	Master’s degree	26	8.31%
	Doctor’s degree or above	14	4.47%
Income	<3,000	113	36.10%
	3,000–4,999	98	31.31%
	5,000–7,999	71	22.68%
	8,000–11,999	26	8.31%
	≧12,000	5	1.60%
Physical condition	Very good	106	33.86%
	Relatively good	122	38.98%
	General	80	25.56%
	Relatively poor	5	1.60%
	Very bad	0	0

### Model of measurement

Cronbach’s alpha coefficient and combined reliability were used in this study to test the consistency of the scale. From Table 3, we can see that the overall Cronbach’s alpha coefficient and combined reliability values were both above 0.7, indicating good internal consistency for each dimension of the scale.

**Table 3:** Reliability and validity test.

Items	Cronbach’s Alpha	Rho_A	CR	AVE
DC	0.915	0.916	0.937	0.747
DPR	0.953	0.954	0.964	0.842
ED	0.941	0.941	0.955	0.810
MC	0.938	0.938	0.953	0.801
MP	0.921	0.922	0.940	0.760
MQ	0.926	0.929	0.944	0.772
PS	0.955	0.955	0.965	0.847
SU	0.936	0.941	0.952	0.797

Doctor-patient communication (DC); Doctor-patient relationship (DPR); Emotional dependence (ED); Healthcare costs (MC); Healthcare process (MP); Perception of healthcare quality (MQ); Patient satisfaction (PS); Online healthcare community use (SU).

Hair et al. (41) suggested that the following indicators be used to test whether the measurement model has good convergent validity: (1) standardized factor load >0.7; (2) CR (composite reliability) > 0.7; AVE (average variance extracted) > 0.5. As can be seen in Table 4, the standardized factor load of all measurement items was greater than 0.7. Table 3 shows that the variation range of CR was from 0.937 to 0.965, and AVE ranged from 0.747 to 0.847, indicating that the measurement model in this study had high convergent validity.

**Table 4:** Cross loadings.

Items	DC	DPR	ED	MC	MP	MQ	PS	SU
DC1	0.862							
DC2	0.846							
DC3	0.858							
DC4	0.908							
DC5	0.845							
DPR1		0.889						
DPR2		0.926						
DPR3		0.934						
DPR4		0.948						
DPR5		0.890						
ED1			0.886					
ED2			0.934					
ED3			0.917					
ED4			0.909					
ED5			0.853					
MC1				0.903				
MC2				0.882				
MC3				0.914				
MC4				0.890				
MC5				0.886				
MP1					0.835			
MP2					0.915			
MP3					0.905			
MP4					0.862			
MP5					0.839			
MQ1						0.890		
MQ2						0.884		
MQ3						0.908		
MQ4						0.87		
MQ5						0.840		
PS1							0.929	
PS2							0.93	
PS3							0.948	
PS4							0.917	
PS5							0.876	
SU1								0.868
SU2								0.865
SU3								0.939
SU4								0.891
SU5								0.899

Discriminant validity analysis is used to verify whether there are statistically significant differences between different research dimensions. Theoretically, measurement items at different dimensions should not be highly correlated. If the correlation coefficient is higher than 0.85, the contents measured by these measurement items belong to the same dimension (42). Both Fornell-Larcker criterion and HTMT method were used to test the discriminant validity. The discriminant validity values of Fornell-Larcker criterion are shown in Table 5. The data present Pearson correlation coefficients between variables, where the diagonal line represents the square root value of AVE. When Pearson’s correlation coefficient between two variables is less than the square sum value of the two variables AVE, this indicates that the discriminant validity between two variables is acceptable. All dimensions in this study met these requirements, indicating that the discriminative validity of each dimension was acceptable. On the other hand, this paper also used the HTMT approach for discriminant validity, the HTMT value was 0.791, lower than the suggested value (0.85), indicating good discriminant validity.

**Table 5:** Discriminative validity.

Items	DC	DPR	ED	MC	MP	MQ	PS	SU
DC	**0.864**							
DPR	0.529	**0.918**						
ED	0.580	0.578	**0.900**					
MC	0.549	0.501	0.611	**0.895**				
MP	0.598	0.276	0.588	0.677	**0.872**			
MQ	0.581	0.639	0.648	0.597	0.574	**0.878**		
PS	0.572	0.601	0.630	0.606	0.561	0.604	**0.920**	
SU	0.601	0.397	0.397	0.418	0.447	0.411	0.420	**0.893**

The diagonal (bold) elements are the square roots of AVEs, and off-diagonal elements are correlations between constructs.

### Common method bias

Harman’s single-factor test was used to test the data for common method bias (43) and ensure the accuracy and objectivity of the analysis results. SPSS22.0 software was to conduct exploratory factor analysis for all other variables after the control variables were eliminated, and then the unrotated factor variance explanation rate was assessed through principal component analysis. The results showed that the total variance of the first principal component interpretation was 25.029%, which was far less than the critical value of 50% (44), indicating that no excess variance was present, and there was no common method bias.

## Model of structure

### Structural equation model results

The degree of matching of the research model is shown in Table 6, and the model indicators all meet fitting criteria, indicating that the construction and fitting results of the model were ideal.

**Table 6:** Model matching degree.

Variable	Model of saturation	Model of estimation
SRMR	0.051	0.158
d_ULS	2.143	20.490
d_G	1.619	2.485
Chi-square value	2658.508	3287.440
NFI	0.833	0.794

**Figure 2:**
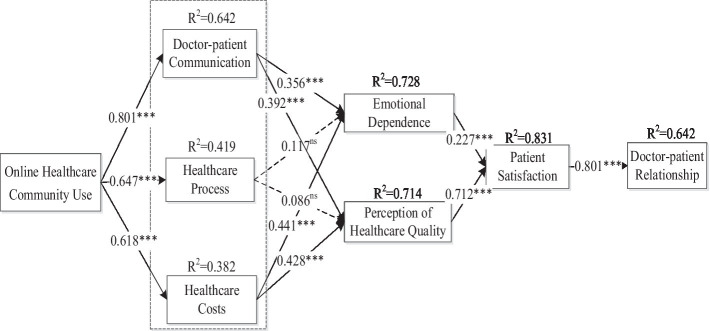
Structural equation model results.

The structural equation model results for this study are shown in Figure 2. Online healthcare community had a positive impact on doctor-patient communication (β = 0.801, *p* < 0.001), healthcare treatment process (β = 0.647, p < 0.001), and healthcare treatment cost (β = 0.618, *p* < 0.001). Therefore, H1, H2, and H3 are considered to be supported. Second, doctor-patient communication had a positive impact on patients’ emotional dependence (β = 0.356, *p* < 0.001) and patients’ perception of healthcare quality (β = 0.392, *p* < 0.001). Therefore, H4 and H5 were supported. Healthcare procedures had no significant effects on patients’ emotional dependence (β = 0.119, *p* > 0.05) or patients’ perception of healthcare quality (β = 0.086, *p* > 0.05). Therefore, hypotheses H6 and H7 were not supported.

Healthcare costs had a positive impact on patients’ emotional dependence (β = 0.441, *p* < 0.001) and patients’ perception of healthcare quality (β = 0.428, *p* < 0.001). Therefore, H8 and H9 were supported. Finally, affective dependence had a positive effect on patient satisfaction (β = 0.227, p < 0.001), so H10 was supported. Patients’ perceptions of healthcare quality had a positive impact on patients’ satisfaction (β = 0.712, *p* < 0.001). Therefore, H11 was supported. Finally, patient satisfaction had a positive effect on the doctor-patient relationship (β = 0.801, *p* < 0.001). Therefore, H12 was supported (see Table 7).

**Table 7:** Model results.

The path	Initial sample (O)	Initial sample(M)	STDEV	T(|O/STDEV|)	*P*
DC → ED	0.356	0.354	0.084	4.224	0
DC → MQ	0.392	0.394	0.082	4.788	0
ED → PS	0.227	0.232	0.07	3.232	0.001
MC → ED	0.441	0.439	0.093	4.755	0
MC → MQ	0.428	0.423	0.075	5.719	0
MP → ED	0.117	0.123	0.107	1.093	0.275
MP → MQ	0.086	0.09	0.096	0.898	0.37
MQ → PS	0.712	0.708	0.064	11.1	0
PS → DPR	0.801	0.802	0.041	19.522	0
SU → DC	0.801	0.803	0.04	20.106	0
SU → MC	0.618	0.619	0.057	10.771	0
SU → MP	0.647	0.651	0.059	10.905	0

### Mediation effect

#### Mediating effects of doctor-patient communication, healthcare process, and healthcare cost

Bootstrapping was used to test for mediating effects (45), in particular for doctor-patient communication, healthcare process, and healthcare cost. As shown in Table 8, first, doctor-patient communication has a full mediating effect between the use of online healthcare community and emotional dependence. This indicates that the use of online healthcare communities completely mediated patients’ emotional dependence through doctor-patient communication. Doctor-patient communication had a fully mediating effect between the use of online healthcare community and patients’ perception of healthcare quality. This suggests that the use of online healthcare communities affects patients’ perception of healthcare quality completely through doctor-patient communication.

**Table 8:** Mediating effects of doctor-patient communication, healthcare process and healthcare cost.

IV	M	DV	IV-DV	IV-M	(IV + M-DV)		Effect
					IV	M	
SU	DC	ED	0.600**	0.811**	−0.078	0.841**	Full
SU	DC	MQ	0.614**	0.811**	−0.043	0.815**	Full
SU	MP	ED	0.600**	0.648**	0.151**	0.691**	Partial
SU	MP	MQ	0.614**	0.648**	0.191**	0.651**	Partial
SU	MC	ED	0.600**	0.619**	0.157**	0.715**	Partial
SU	MC	MQ	0.614**	0.619**	0.194**	0.678**	Partial

*p < 0.05, **p < 0.01.

Second, the healthcare process has a partial mediating effect between the use of the online healthcare community and emotional dependence. On the one hand, the use of online healthcare community affects patients’ emotional dependence; On the other hand, it also affects patients’ emotional dependence by affecting the healthcare process. The healthcare process has a partially mediating effect between the use of online healthcare community and patients’ perception of healthcare quality. On the one hand, the use of online healthcare community affects patients’ perception of healthcare quality, but on the other, the use of online healthcare communities affects patients’ perceptions of healthcare quality by affecting the healthcare process.

Third, healthcare cost has a partial mediating effect between the use of online healthcare community and emotional dependence. On the one hand, the use of online healthcare community affects patients’ emotional dependence; on the other hand, it also affects patients’ emotional dependence by affecting healthcare costs. Healthcare costs have a partially mediating effect between the use of online healthcare community and patients’ perception of healthcare quality. On the one hand, the use of online healthcare community affects patients’ perception of healthcare quality, but on the other, the use of online healthcare communities affects patients’ perception of healthcare quality by affecting healthcare costs.

#### Mediating effects of emotional dependence and patients’ perception of healthcare quality

Bootstrapping was used to test the mediating effects of emotional dependence and patients’ perception of healthcare quality. As shown in Table 9, studies show that, first, emotional dependence has a partially mediating effect between doctor-patient communication and patient satisfaction. This indicates that on the one hand, doctor-patient communication affects patient satisfaction; on the other hand, doctor-patient communication affects patient satisfaction by influencing emotional dependence. Second, emotional dependence has a partially mediating effect between the healthcare process and patient satisfaction. This indicates that on the one hand, the healthcare process affects patient satisfaction; on the other hand, the healthcare process affects patient satisfaction by affecting emotional dependence. Third, emotional dependence has a partially mediating effect between healthcare cost and patient satisfaction. This indicates that on the one hand, healthcare costs affect patient satisfaction; on the other hand, healthcare cost affects patient satisfaction by influencing emotional dependence.

**Table 9:** Mediating effect of emotional dependence and patients’ perception of healthcare quality.

IV	M	DV	IV-DV	IV-M	(IV+ M - DV)		Effect
					IV	M	
DC	ED	PS	0.776**	0.787**	0.323**	0.575**	Partial
MP	ED	PS	0.762**	0.790**	0.281**	0.608**	Partial
MC	ED	PS	0.806**	0.812**	0.388**	0.515**	Partial
DC	MQ	PS	0.776**	0.787**	0.172**	0.767**	Partial
MP	MQ	PS	0.762**	0.776**	0.150**	0.787**	Partial
MC	MQ	PS	0.806**	0.799**	0.233**	0.718**	Partial

*p < 0.05, **p < 0.01.

On the other hand, patients’ perception of healthcare quality has a partial mediating effect between doctor-patient communication and patient satisfaction. This indicates that, on the one hand, doctor-patient communication affects patient satisfaction; on the other hand, doctor-patient communication affects patient satisfaction by affecting the perception of healthcare quality. Second, the perception of healthcare quality has a partial mediating effect between the healthcare treatment process and patient satisfaction. This indicates that on the one hand, the healthcare process affects patient satisfaction; on the other hand, the healthcare process affects patient satisfaction by affecting the perception of healthcare quality. Third, the perception of healthcare quality has a partially mediating effect between healthcare cost and patient satisfaction. This indicates that on the one hand, healthcare cost affects patient satisfaction; on the other hand, healthcare cost affects patient satisfaction by affecting the perception of healthcare quality.

#### Mediating effect of patient satisfaction

Bootstrapping was also used to test the mediating effects of patient satisfaction (40). As can be seen from Table 10, first, patient satisfaction has a partially mediating effect between emotional dependence and the doctor-patient relationship. This indicates that emotional dependence affects the doctor-patient relationship; on the other hand, emotional dependence affects the doctor-patient relationship by affecting patient satisfaction. Second, patient satisfaction has a partially mediating effect between the perception of healthcare quality and doctor-patient relationship. On the one hand, the perception of healthcare quality affects the doctor-patient relationship; on the other hand, perceived healthcare quality affects the doctor-patient relationship by influencing patient satisfaction.

**Table 10:** Analysis of the mediating effect of patient satisfaction.

IV	M	DV	IV-DV	IV-M	(IV+ M - DV)		The effect
					**IV**	**M**	
ED	PS	DPR	0.779**	0.830**	0.364**	0.499**	Partial
MQ	PS	DPR	0.843**	0.904**	0.635**	0.228**	Partial

*p < 0.05, **p < 0.01.

## Conclusion

### Key findings

The results of hypothesis testing are summarized in Table 11 below. The results show that the use of an online healthcare community has a positive impact on doctor-patient communication, improvement of healthcare procedures, and reduction of healthcare costs. In addition, doctor-patient communication has a positive impact on patients’ emotional dependence and patients’ perception of healthcare quality. However, no significant positive effect was seen on patients’ emotional dependence and perceived healthcare quality. The cost of healthcare treatment has a positive effect on patients’ emotional dependence and perception of healthcare quality. Patients’ emotional dependence has a positive effect on patients’ satisfaction. Patients’ perception of healthcare quality has a positive impact on patients’ satisfaction. Finally, patient satisfaction has a positive impact on the doctor-patient relationship.

**Table 11:** Summary of hypothesis testing results.

Hypotheses		Supported
H1	The use of online healthcare communities has a positive impact on doctor-patient communication.	Yes
H2	The use of online healthcare community has a positive impact on the improvement of healthcare treatment process.	Yes
H3	The use of online healthcare community has a positive impact on reducing healthcare costs.	Yes
H4	Doctor-patient communication has a positive impact on patients’ emotional dependence.	Yes
H5	Doctor-patient communication has a positive impact on patients’ perception of healthcare quality.	Yes
H6	Improvement of healthcare procedures have a positive impact on patients’ emotional dependence.	No
H7	Improvement of healthcare treatment process has a positive impact on patients’ perception of healthcare quality.	No
H8	Improvement of healthcare costs have a positive impact on patients’ emotional dependence.	Yes
H9	Improvement of healthcare costs have a positive impact on patients’ perception of healthcare quality.	Yes
H10	Patient emotional dependence has a positive effect on patient satisfaction.	Yes
H11	Patients’ perception of healthcare quality has a positive impact on patient satisfaction.	Yes
H12	Patient satisfaction has a positive impact on the doctor-patient relationship.	Yes

This part discusses the process and mechanisms of the influence of online healthcare community on the doctor-patient relationship. The findings of this study, drawing on a large-sample survey in China, are as follows:

First, the use of online healthcare community has a positive impact on doctor-patient communication, healthcare process, and healthcare costs. Previous studies mainly focused on the influencing factors of patients’ healthcare choice behaviors in online healthcare communities (46), the influencing factors of user acceptance and use behavior in online health community, the influencing factors of user satisfaction in an online health community, doctor-patient participation in online healthcare community and influencing factors (15, 26). This study added the further findings that the use of online healthcare community can affect doctor-patient communication, healthcare process, and healthcare cost.

Second, the study found that doctor-patient communication had a positive impact on patients’ emotional dependence and perceptions of healthcare quality. This indicates that doctor-patient communication through an online healthcare community has a significant impact on improving patients’ emotional dependence and perception of healthcare quality. Previous studies largely focused on the impact of healthcare service quality, negative emotions (47), patient perceptions and other factors on the perception of healthcare quality (48). From existing studies, this study further found that the use of the online healthcare community would affect patients’ emotional dependence and perception of healthcare quality. Second, the effect of the healthcare process on patients’ emotional dependence and perception of healthcare quality was not significant. One of the possible reasons may be that emotional dependence and perception of healthcare quality mainly depend on the interaction between doctors and patients (3, 9), healthcare process mainly focused on the improvement of the medical process, such as reducing the cost of queuing time. Therefore, the healthcare process has no significant influence on them. Finally, the cost of healthcare has a positive impact on patients’ emotional dependence and perception of healthcare quality. This indicates that with the reduction of healthcare costs, patients’ emotional dependence and perception of healthcare quality will gradually improve.

Third, emotional dependence has a positive effect on patient satisfaction. This indicates that with the increase in patients’ emotional dependence, their perception of healthcare quality level will gradually improve, and their satisfaction will be significantly improved. Previous studies focused on social capital (49), network media communication (13, 33) and so on. This study additionally showed that emotional dependence has a positive impact on patient satisfaction. Second, patients’ perception of healthcare quality has a positive impact on patient satisfaction. This indicates that with the increase in patients’ perception of healthcare quality, their perception of healthcare quality is significantly improved, and their satisfaction is also significantly improved. Finally, studies have shown that patient satisfaction has a positive impact on the doctor-patient relationship. This indicates that with the improvement of patient satisfaction, the doctor-patient relationship can be improved.

### Theoretical implications

First, this paper forms a contribution to the doctor-patient relationship literature by investigating the relationship between online healthcare community use and the doctor-patient relationship. Most prior research has focused on patients’ behavior, such as their willingness to engage in a community (18), their informational and emotional support (48), social support (50), their knowledge sharing (51) and information seeking (52), their information disclosure intention (53); physicians’ behavior, such as group joining behavior (54), and physicians’ teams’ performance (55); differences in demographic characteristics (56), and value co-creation behaviors in online health communities (47). However, few studies have focused on the relationship between online healthcare community use and the doctor-patient relationship. To fill this gap in the research, this paper investigates the relationship between the use of an online healthcare community and the doctor-patient relationship. The conclusions of this research enrich the understanding of the use of the online healthcare community and the relationship between doctors and patients.

Second, this paper contributes to the literature on the doctor-patient relationship by revealing the mediation process between online healthcare community use and the doctor-patient relationship. In the limited range of literature, Bernardi and Wu (46), drawing on 44 semi-structured interviews with members of an online health community for people with diabetes, find that patients exercise a great deal of agency in evaluating healthcare options not only by activating the logic of personal choice but also by appropriating the logic of medical professionalism. However, their study is limited in the types of results it can generate, as collects qualitative data, without relevant empirical tests, and does not reveal the intermediary process of the impact of the online healthcare community on the doctor-patient relationship. Unlike previous studies, this study found that online healthcare community usage affects the doctor-patient relationship by improving doctor-patient communication, improving healthcare procedures, and reducing healthcare costs, all of which further affect patients’ emotional dependence and patients’ perception of healthcare quality, finally affecting doctor-patient relationship through the mediator of patients’ satisfaction. This study clarifies the process mechanism of the impact of online healthcare community use on the doctor-patient relationship.

### Practical implications

This paper has practical and guiding significance for doctors, patients, and administrators of the online healthcare community. First, this paper provides guidance and suggestions to enable doctors and patients to use online healthcare communities more actively and to platform operators to promote doctors and patients in this type of effective use. Drawing on online and offline aspects, this paper studies the influencing factors of patients’ use of online healthcare community, putting forward strategies to promote patients’ active use of online healthcare community and providing corresponding suggestions for doctors to make better use of online healthcare community to relieve shortages of offline healthcare resources and guide patients to use online healthcare communities. This paper also puts forward suggestions for the online healthcare community platform operators to improve the platform operation mechanism and attract more doctors and patients.

Second, doctors should strengthen doctor-patient communication, patiently listening to patients’ views on their own health, supporting and encouraging patients to participate in the healthcare decision-making process and adapting to the transformation of the healthcare service mode from a doctor-led mode to a patient-centered one. At the same time, clinicians should also pay attention to the evaluation of patients throughout the network, carry forward the advantages, improve the shortcomings, and establish an all-around personal brand, stimulating the participation of doctors. On the other hand, through an online healthcare community, patients can carry out health self-examination and real-time monitoring of their own health status, to reach healthcare services in good time. After seeing a doctor, patients can use the online platform to reflect on their own recovery, pose questions, and effectively carry out post-service activities. Thus, a smooth communication channel can be formed between doctors and patients, and the doctor-patient contradictions and disputes caused by information asymmetry can be effectively avoided, thus promoting the healthy development of the doctor-patient relationship.

Third, the Chinese government has proposed to promote the “Internet + healthcare” consultation model to meet growing healthcare and health needs in the country. Therefore, it will be necessary to analyze the factors influencing the use of the online healthcare community and the use effect of the platform, to support Chinese government organs, healthcare organizations at all levels, and operators of online healthcare community platforms to continually improve the specific implementation suggestions on the standardized use of “Internet + healthcare.” This study focuses on the use of online healthcare communities and emphasizes the importance of online healthcare communities in relieving the shortage of healthcare resources and improving the doctor-patient relationship. This will help Chinese governmental organizations, healthcare organizations at all levels, and online healthcare community platforms formulate detailed guidelines for promoting the use of online healthcare communities.

Finally, for government organizations, administrative departments should strengthen the supervision of healthcare and health websites in the system, and crack down on and rectify websites that spread false health information and malicious healthcare products. Relevant laws and regulations are constantly being supplemented and improved to ensure the scientific nature and accuracy of Internet healthcare and healthcare information, promoting the healthy and orderly development of online healthcare and online healthcare information services. On the other hand, industry organizations such as the Chinese Healthcare Association and Healthcare Doctor Association regularly have healthcare experts review and evaluate various online healthcare platforms, publishing these reviews to the public, to allow patients to correctly identify healthcare network information and identify high-quality Internet healthcare services. In addition, all healthcare units, especially public hospitals, should take the initiative to fulfill the social responsibility of popularizing healthcare knowledge and improving residents’ healthcare literacy, developing apps specifically for popularizing healthcare knowledge among patients, equipped with functions such as self-detection of health status, introducing common disease symptoms, conditions, diagnoses, and treatments, as well as guidance for rehabilitation programs. To help patients clarify their subjective cognition about the disease and reduce the large gap between the expected diagnosis and treatment effect and the actual diagnosis and treatment effect of patients, not only to promote the promotion and application of online healthcare community but also to help ease the tense doctor-patient relationship.

### Limitations and directions for future research

Although the findings of this study are valid and valuable, they should be approached with caution for several reasons. First and most importantly, the sample may not represent the whole population of patients in China. Due to time and data constraints, 313 patients’ data were used for analysis. Future studies can test the generalizability of this study by collecting additional patient data. Second, online healthcare community use is a subjective factor across patients, which makes it inevitable that there will be some self-report bias in the measurement. In addition, respondents’ external environment, other interference, and respondents own emotions naturally had an impact on the quality of the questionnaire results. Therefore, future research can combine text mining, emotion analysis, and other methods to collect information related to patients’ healthcare search and consultation in an online healthcare community, to enrich research data sources, and analyze the impact of the use of online healthcare communities on the doctor-patient relationship through the combination of subjective and objective data. Finally, this article only explores the mechanism of its effect on the doctor-patient relationship from the perspective of the online healthcare community, although the doctor-patient relationship may also be affected by other external factors, such as relevant national healthcare and social security policies, laws and regulations, patient disease types, healthcare staff income, and treatments (20). Future research should fully consider the impact of external factors on the doctor-patient relationship to conduct a more scientific and thorough systematic analysis and in-depth assessment of the doctor-patient relationship.

## Data availability statement

The original contributions presented in the study are included in the article/Supplementary material, further inquiries can be directed to the corresponding author.

## Author contributions

XZ and YZ: conceptualization. ZW: writing—original draft. DH: investigation. FH: writing—review and editing. LM: model analysis. All authors have read and agreed to the published version of the manuscript.

## Funding

This research was funded by the Social Science Planning Project of Shandong Province (19CDNJ06 and 20CJJJ21), Development plan of the Youth Innovation Team in colleges and universities of Shandong Province (2021RW029), and Shandong Institute of Management 2021 Scientific Research Launch Plan Project (Project No. QH2021R15). 

## Conflict of interest

The authors declare that the research was conducted in the absence of any commercial or financial relationships that could be construed as a potential conflict of interest.

## Publisher’s note

All claims expressed in this article are solely those of the authors and do not necessarily represent those of their affiliated organizations, or those of the publisher, the editors and the reviewers. Any product that may be evaluated in this article, or claim that may be made by its manufacturer, is not guaranteed or endorsed by the publisher.
